# Altering translation allows *E. coli* to overcome G-quadruplex stabilizers

**DOI:** 10.1093/nar/gkaf264

**Published:** 2025-04-07

**Authors:** Rachel R Cueny, Andrew F Voter, Aidan M McKenzie, Marcel Morgenstern, Kevin S Myers, Michael M Place, Jason M Peters, Joshua J Coon, James L Keck

**Affiliations:** Biomolecular Chemistry Department, School of Medicine and Public Health, University of Wisconsin-Madison, Madison, WI, 53706 United States; Biomolecular Chemistry Department, School of Medicine and Public Health, University of Wisconsin-Madison, Madison, WI, 53706 United States; Biomolecular Chemistry Department, School of Medicine and Public Health, University of Wisconsin-Madison, Madison, WI, 53706 United States; Biomolecular Chemistry Department, School of Medicine and Public Health, University of Wisconsin-Madison, Madison, WI, 53706 United States; National Center for Quantitative Biology of Complex Systems, University of Wisconsin-Madison, Madison, WI, 53706 United States; Great Lakes Bioenergy Research Center and the Wisconsin Energy Institute, University of Wisconsin-Madison, Madison, WI, 53726 United States; Great Lakes Bioenergy Research Center and the Wisconsin Energy Institute, University of Wisconsin-Madison, Madison, WI, 53726 United States; Pharmaceutical Sciences Division, School of Pharmacy, University of Wisconsin-Madison, Madison, WI, 53706 United States; Biomolecular Chemistry Department, School of Medicine and Public Health, University of Wisconsin-Madison, Madison, WI, 53706 United States; National Center for Quantitative Biology of Complex Systems, University of Wisconsin-Madison, Madison, WI, 53706 United States; Department of Chemistry, University of Wisconsin-Madison, Madison, WI, 53706 United States; Morgridge Institute for Research, Madison, WI, 53715 United States; Biomolecular Chemistry Department, School of Medicine and Public Health, University of Wisconsin-Madison, Madison, WI, 53706 United States

## Abstract

G-quadruplex (G4) structures can form in guanine-rich DNA or RNA and have been found to modulate cellular processes, including replication, transcription, and translation. Many studies on the cellular roles of G4s have focused on eukaryotic systems, with far fewer probing bacterial G4s. Using a chemical-genetic approach, we identified genes in *Escherichia coli* that are important for growth in G4-stabilizing conditions. Reducing levels of translation elongation factor Tu or slowing translation initiation or elongation with kasugamycin, chloramphenicol, or spectinomycin suppress the effects of G4-stabilizing compounds. In contrast, reducing the expression of specific translation termination or ribosome recycling proteins is detrimental to growth in G4-stabilizing conditions. Proteomic and transcriptomic analyses reveal decreased protein and transcript levels, respectively, for ribosome assembly factors and proteins associated with translation in the presence of G4 stabilizer. Our results support a model in which reducing the rate of translation by altering translation initiation, translation elongation, or ribosome assembly can compensate for G4-related stress in *E. coli*.

## Graphical Abstract

**Graphical Abstract:**
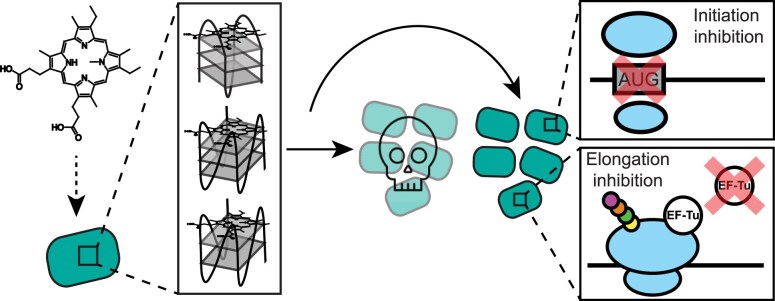

## Introduction

G-quadruplexes (G4s) are nucleic acid structures that can fold in guanine-rich stretches of DNA or RNA [1]. The structures comprise stacked G-tetrads (four guanines hydrogen bonded to one another) surrounding a core of monovalent cations. Interest in possible biological roles for G4s emerged upon discovering that guanine-rich telomeres in humans and other eukaryotes could fold into G4 structures [2–6].

G4s have been found to modulate replication, transcription, and translation processes. For example, G4s can act as barriers to DNA replication in eukaryotes, an effect that is exacerbated upon depletion of accessory DNA helicases [7–10]. DNA G4s can also impact transcription in a strand- and location-dependent manner [11–14]. G4s found in mRNA transcripts can regulate translation efficiency, with G4s in open reading frames decreasing translation and G4s in untranslated stimulating or impeding translation in eukaryotes and prokaryotes [1, 11, 15–18]. Several proteins that bind and unwind G4s have been shown to govern G4 homeostasis in cells [1, 19–26].

The impact of G4s in neurodegenerative disorders, oncogenes, and telomeres has motivated studies to understand G4s in eukaryotes [2, 12, 13, 17, 27–29]. Despite advances in understanding the roles of G4s in eukaryotic systems, parallel studies in bacteria have been far more limited. One notable exception is from the pathogenic bacteria *Neisseria gonorrhoeae*, in which a G4 is essential for antigenic variation [30, 31]. Beyond this example, most studies investigating G4s in *Escherichia coli* and other bacteria have focused on identifying potential quadruplex forming sequences or have investigated the effects of non-native G4 sequences in plasmids on growth or gene expression [1, 11, 32, 33]. Other studies have taken a candidate-based approaches to examine the roles of selected DNA helicases or DNA repair proteins in G4 processing *in vitro* or *in vivo* [1, 23, 26].

To better understand the challenges presented by G4s in bacteria, we carried out tandem chemical-genetic screens to identify genes that are important for *E. coli* growth in the presence of the G4-stabilizing compound N-methyl-mesoporphyrin IX (NMM). A transposon-sequencing (Tn-seq) screen showed that transposon insertions were strongly selected in two genes, *tufA* and *tufB*, in cells grown under G4-stabilizing conditions. *tufA::kan* and *tufB::kan* mutations suppressed NMM growth sensitivity in *E. coli* and, to a lesser extent, sensitivity to a structurally-distinct G4 stabilizing compound (Braco-19), consistent with the mutations impacting cellular response to stabilized G4s. *tufA* and *tufB* encode for the same protein, elongation factor (EF) Tu, which escorts charged tRNAs to the ribosome during translation and impacts the rates of translation and cell growth [34–36]. Disruption of either *tufA* or *tufB* reduced cellular EF-Tu levels, suggesting that slowing translation could counter the adverse effects of G4 stabilization. In concurrence with this idea, low doses of the bacterial translation inhibitors chloramphenicol, spectinomycin, and kasugamycin each improved the growth of *E. coli* in the presence of NMM. A CRISPR interference (CRISPRi) screen identified the importance of a ribosome release factor (RF1) and an EF involved in ribosome recycling (EF-G) for growth in NMM, further supporting a model in which translation factors are linked to overcoming stabilized G4s *E. coli*. Proteomic and transcriptomic analyses identified several proteins and transcripts that were differentially expressed ± NMM, with ribosome assembly/biogenesis genes often being downregulated in cells grown in the presence of the G4 stabilizer. Our observations collectively support a model in which stabilized G4s influence translation, and alterations to translation initiation and elongation can allow *E. coli* to overcome the detrimental impacts of stabilized G4s.

## Materials and methods

### Strain construction

Unless otherwise specified, all cells used in this study are derived from *E. coli* MG1655. To generate *E. coli* knockout strains, P1 transductions were carried out using Keio collection strains as donor strains [37, 38]. P1 phage lysates grown on Keio collection donor strains were used to transduce the MG1655 strains or CRISPRi strains [39] (to make *tolC* knockout of selected CRISPRi strains), which were sensitive to kanamycin. To validate strains, transductions were grown on LB plates supplemented with 50 μg/mL kanamycin and screened using colony PCR to validate the proper insertion of the kanamycin resistance cassette. To remove the kanamycin-resistant cassette from MG1655 *tolC::kan* to enable additional P1 transductions in this strain, MG1655 *tolC::kan* electrocompetent cells were generated and transformed with a plasmid encoding the FLP recombinase (pCP20) [40]. Cells were recovered at 30°C and grown overnight on Super Optimal Broth (SOB) plates supplemented with 100 μg/mL ampicillin at 30°C. Single colonies from the plate were then grown overnight in LB at 43°C to promote loss of the temperature-sensitive plasmid. A 10^−6^ dilution of cells was grown on LB plates at 30°C overnight to obtain individual colonies, which were then streaked onto LB only, LB supplemented with 100 μg/mL ampicillin, and LB supplemented with 50 μg/mL kanamycin. Colonies that only grew on the LB without antibiotic plates were selected as MG1655 Δ*tolC* cells and utilized for downstream applications.

### Transposome preparation and transposition

Transposome preparation was carried out as previously described [41, 42]. The EZ-Tn5 < DHFR-1 > transposon kit (Epicentre) and the E54K/M56A/L372P Tn5 hyperactive variant transposase were used for transposon mutagenesis. The Tn5 transposon was amplified using Phusion polymerase (NEB) and oligonucleotide oAM054. Transposase purification was carried out as previously described [41, 43]. Transposomes were prepared by incubating 2.5 pmol Tn5 DNA with 0.5 nmol Tn5 transposase for 3 hours at ambient temperature and then dialyzed into 1x TE buffer before electroporation.

Electrocompetent *E. coli* cells were generated as previously described [41]. Briefly, *E. coli* was grown at 37°C to an OD_600_ ∼0.4 and cooled at 4°C for an hour. Cells were centrifuged at 10 750 rcf, and pellets were washed in 10% glycerol three times. Cells were then resuspended in 2 mL 10% (v/v) glycerol, 0.125% (w/v) yeast extract, and 0.25% (w/v) tryptone media before flash freezing and storing electrocompetent cells at −80°C. Five μL of transposome was combined with 100 μL of electrocompetent cells, electroporated, and recovered in 1 mL of SOC media at 37°C for 1 h. Cells were plated on SOB-agar supplemented with 10 μg/mL trimethoprim to select for cells containing transposon insertions. Transposon mutants (∼200 000 colonies) were pooled from plates using 2 mL of LB to scrape colonies off plates and then stored in 50% glycerol at −80°C.

### Selection of tolerated transposon mutations in G4-stabilizing conditions

For the pilot Tn-seq experiment (Supplementary Fig. S1), a Tn5 transposase generated library in MG1655 *sulB103* was utilized [42]. This library was diluted from a glycerol stock 1:10 000 in fresh LB, and 250 μL of dilution was plated on SOC plates and SOC plates supplemented with 10 μM NMM. NMM is readily taken up by *E. coli* [26]. Plates were grown overnight at 37°C, and there were estimated ∼100 000 colonies grown on SOC alone and ∼150 000 colonies grown on NMM-supplemented plates. Colonies were pooled with LB, samples were diluted to an OD_600_ of ∼4.0, and 1 mL of concentrated cells underwent genomic DNA preparation using the Wizard Genomic DNA Purification Kit (Promega). DNA was quantified using the QuantiFluor ONE dsDNA System (Promega). Genomic DNA underwent shearing to ∼200 bp fragments via sonication, and the gDNA fragments were prepared for sequencing using the NEBNext Ultra II DNA Library Prep Kit for Illumina (NEB). Bead-based size selection was employed to enrich for 200 bp fragments and the fragments then underwent a 17-cycle splinkerette PCR using oAM055 as the forward primer and either oAM068 (control) or oAM069 (NMM selected) as the reverse primer for barcoding and multiplexing [42]. An additional bead-based size selection was used to clean up the sample before sequencing at the University of Michigan with a MiSeq platform. Primers oAM058 and oAM059 were used as unique sequencing primers for the control and NMM-treated conditions, respectively.

### Transposon sequencing with Δ*tolC* cells

For the subsequent Tn-seq experiment utilizing an MG1655 Δ*tolC* strain, transposomes were prepared as described above, and the library was prepared as before (except using 1 μg/mL trimethoprim for selection), generating ∼500 000 transposon insertion mutants. MG1655 Δ*tolC* electrocompetent cells were generated as described above.

To select for transposon insertion mutations in control and NMM-treated conditions, libraries were grown on either SOB-agar plates or SOB-agar plates supplemented with 5 μM NMM. To ensure proper coverage after re-selection on plates, ∼1.5 million colonies were collected from the SOB-agar plates, and the SOB-agar plates supplemented with NMM and split into three libraries each. Libraries were passaged a second time in the presence and absence of NMM to generate a second passage library of ∼1.5 million colonies and split into three libraries each.

Libraries were prepared as described in the previous section to prepare DNA for sequencing. Each library was diluted to an OD_600_ of ∼4.0, and 1 mL of concentrated cells underwent genomic DNA preparation using the Wizard Genomic DNA Purification Kit (Promega). DNA was quantified using the QuantiFluor ONE dsDNA System (Promega). Genomic DNA underwent shearing to ∼200 bp fragments via sonication, and the gDNA fragments were prepared for sequencing using the NEBNext Ultra II DNA Library Prep Kit for Illumina (NEB). Bead-based size selection was employed to enrich 200 bp fragments, and the fragments then underwent a 20-cycle splinkerette PCR using a Tn5-enrihcing forward primer (oAM55) and custom reverse primers for multiplexing [42]. A final bead-based size selection was used to select the correct length of DNA. DNA was sequenced at the University of Michigan Advanced Genomics Core using a NextSeq platform (Illumina) with a custom read primer (oAM58) reading the last 10 nt of the transposon. PhiX174 DNA spike was added to the run to ensure sufficient sequence diversity on the flow cell. Then, a custom index read primer (oAM59) and standard Illumina primer were used to sequence the index reads and PhiX174, respectively.

### Data analysis for tn-seq

Tn-seq analysis was done as described previously [42]. Tn-seq sequencing was trimmed with fastx_trimmer.pl version 0.0.13.2 (https://www.hannonlab.org/resources/). The default parameters were used except for the first base to keep (-f flag), which was edited to 10 to remove the transposon sequence. Samples were then split with fastx_barcode_splitter.pl, version 0.013.2 (https://www.hannonlab.org/resources/) using a file that contained the individual barcode sequence and the sample ID, then the barcode was removed from each read in the FASTQ file using Cutadapt version 1.13 [44]. Trimmed FASTQ files were aligned to the *E. coli* K-12 MG1655 genome (NC_000913.3) using Bowtie2, version 1.2 on default parameters [45]. The conditional importance or essentiality of genes was determined using TSAS, version 0.3.0, and Analysis_type2 for 2 sample analyses to compare transposon insertion profiles of NMM-treated cells to cells grown without G4 stabilizer [46]. The weighted reads were determined as previously described [46]. The other parameters were kept at default settings.

### Assessing sensitivity to G4 stabilizers using spot plates

Spot plating experiments to determine sensitivity to G4 stabilizing compounds were carried out as previously described [26]. Briefly, NMM and Braco-19 were prepared by resuspending the compounds in 18 MΩ ultra-pure water, and NMM concentration was assessed using the molar extinction coefficient 145 000 M^−1^ cm^−1^ at 379 nm [47]. NMM and Braco-19 solutions were stored at 4°C. IPTG, kasugamycin, and spectinomycin solutions were made by resuspension in 18 MΩ ultra-pure water and stored at −20°C, and chloramphenicol was resuspended in ethanol and stored at −20°C. G4 stabilizers, IPTG, kasugamycin, spectinomycin, or chloramphenicol were added to LB-agar at the indicated concentrations and stored in the dark. Around 5 mL of each *E. coli* strain were grown overnight and diluted in fresh LB to an OD_600_ ∼1. For spot plating, 10^−1^–10^−6^ dilutions of strains were made in LB, and 10 μL of each dilution was plated onto the LB spot plates (or on M9 minimal medium for M9 spot plates). Spot plates were grown overnight and imaged on the Azure c600. Spot plates were done in triplicate. We note that NMM and Braco-19 were used for two primary reasons. The first is that NMM and Braco-19 are structurally distinct G4 stabilizers—NMM is a porphyrin, whereas Braco-19 is a non-porphyrin. As such, NMM and Braco-19 are appropriate for distinguishing G4-specific effects from compound-specific effects. The second is that NMM and Braco-19 are readily taken up by *E. coli* [26], which rules out cytoplasm exclusion as a reason for differential activities.

### CRISPRi screen

For the CRISPRi screen, strains from the CRISPRi library were grown in plates with 200 μL of LB supplemented with 10 μg/mL chloramphenicol and 4 μL from each glycerol stock of the library. Cells were grown overnight at 37°C and then stored at 4°C overnight. The following day, plates were shaken at 37°C for 5 min, diluted 200-fold into fresh LB, and shaken for 5 min to mix cells. Two μL were plated onto plates with LB-agar alone or LB-agar supplemented with 15 μM NMM, 10 μM IPTG, or 10 μM IPTG and 15 μM NMM together. Plates were grown overnight at 37°C and imaged using the Azure c600 the following day.

### Assessing sensitivity to G4 stabilizers using growth curves

MG1655, Δ*tolC*, Δ*tolC tufA::kan*, and Δ*tolC tufB::kan* cells were grown overnight at 37°C in Luria Broth (LB). The next day, cells were diluted 100-fold into fresh LB and grown to an OD_600_ ∼0.2. Cells were then diluted 100-fold in a 96-well plate either in the presence or absence of NMM, and OD_600_ of the cells was measured every ten minutes over 24 h with continuous shaking at 37°C in a plate reader (BioTek Synergy H1). Growth curves were done in triplicate. The average of each growth condition was then plotted in Prism (10.2.0) with error bars representing the standard error of the mean.

### Western blots

Δ*tolC*, Δ*tolC tufA::kan*, and Δ*tolC tufB::kan* cells overnight cultures were diluted 100-fold in fresh Luria Broth (LB) and grown to an OD_600_ ∼0.3. One mL of cells was pelleted and resuspended in 50 μL of 1x sample buffer (0.8% SDS, 11.5% glycerol, 0.1 M Tris pH 6.8, 0.286 M βME, 0.01% bromophenol blue). Around 5 μL of undiluted sample and 5 μL of sample at various dilutions (1:2, 1:10, or 1:15) were loaded onto a 5–15% PAGE gel (Bio-Rad) and run in 1x SDS running buffer. Proteins were transferred to a nitrocellulose membrane at 4°C for 1.5 h in transfer buffer (25 mM Tris pH 8, 192 mM glycine, 0.03% SDS, 20% methanol). For total protein staining, membranes were incubated with 5 mL total protein stain (LI-COR) for 5 min at ambient temperature on a rocking platform before getting rinsed with wash solution (30% methanol, 6.7% acetic acid) and then imaged on the IR700 channel using the Azure c600. Following total protein stain, the membrane was blocked for 1 h at room temperature in 5% dry milk in 1x PBS (137 mM NaCl, 2.7 mM KCl, 4.3 mM Na_2_HPO_4_, 1.47 mM KH_2_PO_4,_ pH 7.4) and then rinsed with 1x PBS. Membrane was then incubated for 1 h at ambient temperature with anti-EF-Tu antibody (Hycult Biotech) at 2 μg/mL and then rinsed with 1x PBS. Membrane was then incubated with peroxidase conjugated goat anti-mouse antibody (Invitrogen) at 0.1 μg/mL and rinsed with 1x PBS. Using the chemiluminescence setting, the Amersham ECL Prime Western Blotting detection kit was then used to visualize the blot on the Azure c600. The intensity of bands for total protein normalization and EF-Tu blots was carried out using ImageJ and plotted using Prism (10.2.0). Significance was determined in Prism using Welch's two-tailed t-test. Western blots and total protein staining were done in triplicate.

### Proteomics cell growth and cell lysis

Δ*tolC* and Δ*tolC tufA::kan* cells were grown overnight, diluted 100-fold in fresh LB, and grown to an OD_600_ of ∼0.2. Cells were then diluted 100-fold and grown in LB ± 3.5 μM NMM. Cells were grown to an OD_600_ ∼0.2–0.4, then 45 mL of cells were pelleted via centrifugation. The pellets were washed with 5 mL of 1x PBS to remove residual media and then pelleted again. Cell pellets were stored at −80°C.

Cell lysis was initiated by resuspension in 250 μl of lysis buffer (8 M urea and 100 mM Tris pH 8, supplemented with cOmplete™ protease inhibitor cocktail (Roche) according to the manufacturer's specifications). The cell suspension was then subjected to a two-step process to complete lysis: (i) sonication via a probe sonicator for 1 min at medium intensity, followed by a 1 min incubation step on ice. (ii) 250 μl of glass beads (1 mm diameter) were added to each sample, and samples were subjected to four repetitions of the following bead-beating protocol using a Retsch MM400 oscillation mill: 4 min of milling at 30 Hz, followed by a 1 min incubation step on ice. After lysis, samples were subjected to a clarifying spin and protein concentration was determined using a BCA assay (Thermo Pierce). Next, 50 μg of each sample was transferred to a new tube and diluted in lysis buffer to a concentration of 1 mg/mL. To reduce and alkylate cysteine residues, samples were adjusted to 10 mM TCEP and 40 mM chloroacetamide and incubated for 30 min at ambient temperature. Subsequently, samples were diluted in 100 mM Tris pH 8 to a urea concentration of 4 M, followed by adding 1 μg LysC (Wako Chemicals) and a four-hour incubation at ambient temperature. For overnight tryptic digestion at ambient temperature, 50 μg of trypsin (Promega) was added after diluting samples further down to a urea concentration of 1 M. Next morning, the digest was stopped by adjusting samples to 1% TFA, and peptides were purified through Strata-X solid phase extraction cartridges (Phenomenex). Peptide eluates were dried in a vacuum concentrator and resuspended in 0.2% FA to a concentration of 1 mg/ml, ready for mass spectrometry (MS) analysis.

### LC-MS analysis

For Liquid Chromatography-Mass Spectrometry (LC-MS) analysis, the following setup was employed: a Vanquish Neo UHPLC System was coupled to an Orbitrap Astral mass spectrometer via a Nanospray Flex ionization source (all Thermo Scientific), operated at a source voltage of 2 kV. The Vanquish Neo was equipped with a 40 cm fused silica capillary column (75 μm i.d. and  360 μm o.d., Polymicro Technologies) and pulled, etched, and packed in-house using 1.7 μm C18 particles (Waters) as described previously [48]. Individual MS experiments were conducted by separating 1 μg of peptides at a 300 nL/min flow rate at 55°C via a 2 h gradient (Mobile phase A: 0.2% FA, mobile phase B: 0.2% FA, 80% acetonitrile). MS experiments were conducted using a data-dependent acquisition (DDA) regime combining Orbitrap (MS1), and ion trap (MS2) scans under the following parameters: MS1 scans were recorded at a resolution of 240k, a scan range of 300–1350 m/z and a normalized AGC target of 250% with a maximum injection time of 50 ms. MS2 scans were recorded with an isolation window of 0.5 m/z, an HCD collision energy of 23%, at a “Turbo” scan rate speed, a scan range of 150–1350 m/z, and a normalized AGC target of 250%, with a maximum injection time of 14 ms. MIPS, charge state, and dynamic exclusion filters were employed.

### Proteomics data processing and analysis

LC-MS analysis resulted in 12 Thermo RAW files (2 strains x 2 growth conditions x 3 biological replicates), which were processed with MaxQuant, version 2.4.2.0 [49]. MaxQuant was run using the default settings, with the following specifications and changes: i) RAW files were searched against the *E.coli* Uniprot reference proteome (Organism ID: 83 333, downloaded in Sep 2023). All 12 files were searched together but separated into four experiments with three biological replicates per experiment. ii) Under *Group-specific parameters*, LFQ was enabled. Under *Global parameters*, *Min. unique peptides* were set to 1, and *Match between runs* and *iBAQ* were enabled. MaxQuant output files were analyzed via Perseus, version 2.0.11.0 [50]. In Perseus, LFQ intensities were log-transformed, followed by a data filtering step, requiring three out of three valid LFQ intensity values for at least one of the four experiments. Next, missing values were imputed from a normal distribution using Perseus’ default settings. Differences across experiments were then assessed via a two-sided, two-sample t-test. To address the multiple testing problem, a permutation-based false discovery rate calculation based on 250 randomizations was included. Our analysis yielded mean log2 LFQ intensity ratios, *P*-values, and q-values for 2 468 protein groups.

### RNA-seq sample growth and sequencing

Δ*tolC* and Δ*tolC tufA::kan* cells were grown overnight, back diluted 100-fold into fresh LB, and grown to an OD_600_ of ∼0.2. Cells were then diluted 100-fold into 100 mL fresh LB ± 3.5 μM NMM. Cells were then grown to an OD_600_ of ∼0.2–0.4. Cells were harvested via centrifugation at 3214 x g at 4°C for 10 min. Pellets were transferred to 1.7 mL tubes, frozen in LN_2,_ and stored at −80°C.

Pellets were submitted to Genewiz for RNA extraction and library preparation using their RNA-seq with rRNA depletion package. Sequencing was done using the Illumina 2 × 150 bp platform, targeting 20 million paired-end reads per sample. Data analysis was done through Genewiz using DeSeq2 to normalize datasets and generate plots, as shown in Supplementary Fig. S7 [51]. *P*-values were determined via the Wald test *P*-value, and adjusted *P*-values were determined using the Benjamini–Hochberg adjusted *P*-value.

### Sucrose gradients for polysome traces

Δ*tolC* and Δ*tolC tufA::kan* cells were grown in 50 mL of LB overnight. Cultures were then back diluted 100-fold into fresh LB, grown to an OD_600_ of ∼0.2, then diluted 100-fold into 1 L of LB ± 3.5 μM NMM. Cells were then grown to an OD_600_ of ∼0.3–0.5. Cells were harvested via filtration with a 0.45 μm filter (Whatman) and stored at −80°C.

Cells were then lysed via cryomilling in 1 mL of lysis buffer (20 mM Tris pH 8.0, 10 mM MgCl_2_, 100 mM NH_4_Cl, 5 mM CaCl_2_, 100 U/mL DNase I, 1 mM chloramphenicol) at 10 s^−1^ for 1 min three times. Cells were clarified via centrifugation at 20 000 x g at 4°C.

For sucrose gradients, ∼12.5 AU of RNA were loaded onto a 10–50% sucrose gradient (20 mM Tris pH 8.0, 15 mM MgCl_2_, 100 mM NH_4_Cl, 2 mM DTT) prepared using the Biocomp gradient station. Sucrose gradients were ultracentrifuged at 201 000 x g at 4°C for 2.5 h at maximum acceleration and deceleration in the SW 41 Ti rotor. Sucrose gradients were then fractionated on the Biocomp Gradient Station, and A_260_ measurements were monitored. Sucrose gradients were done in triplicate.

To analyze the area under the curve (AUC) corresponding to the small subunit, large subunit, monosomes, and polysomes, the script from [52] was used in RStudio to quantify the fraction of the AUC for each component. This was done for each sucrose gradient, and the AUC percentages were plotted using Prism (10.2.0). *P-*values were determined using Welch’s two-tailed t-test.

## Results

### Disruption of translation genes improves growth in G4-stabilizing conditions

**Figure 1.**
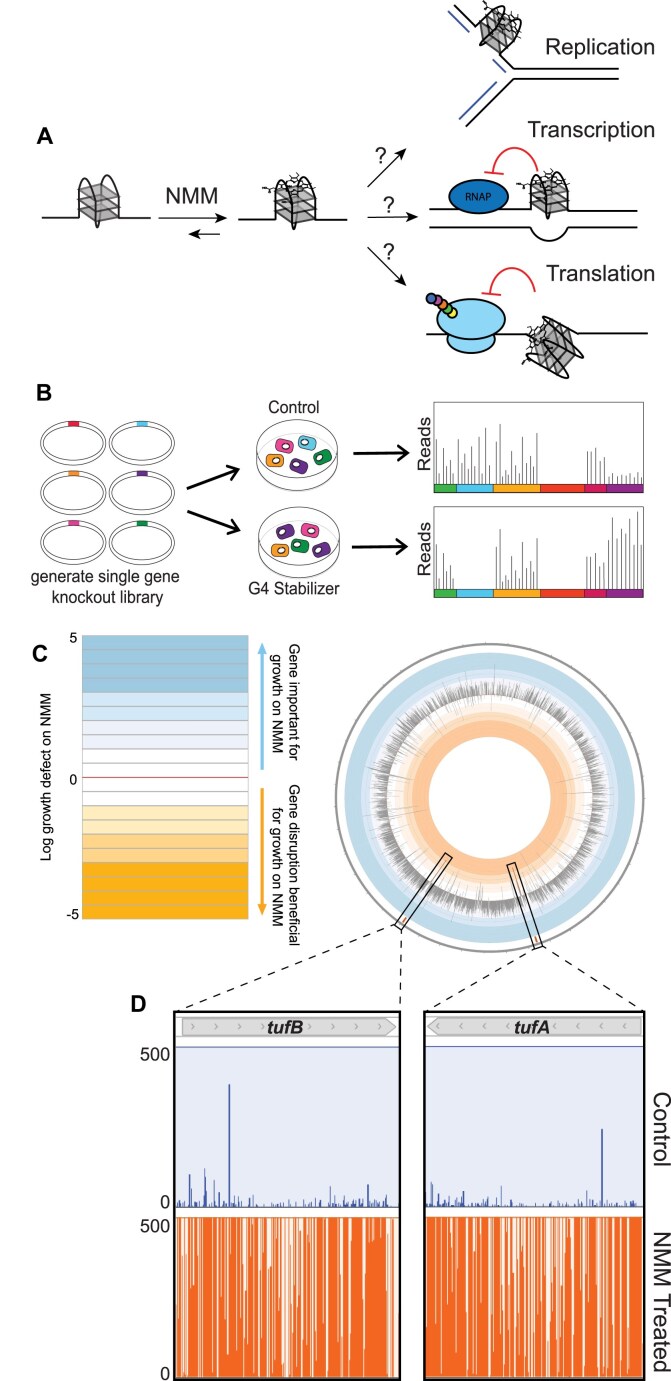
Transposon sequencing reveals genes important for overcoming stabilized G-quadruplexes. (**A**) Cartoon depicting the potential consequences of chemically stabilized G-quadruplexes using NMM. (**B**) Depiction of Tn-seq experiment. A single gene knockout library is generated and grown in control or G4-stabilizing conditions and then sequenced to determine where transposon insertions are tolerated in each condition. (**C**) Circos plot of the log_10_(NMM weighted reads/control weighted reads). (**D**) Zoom-in on insertions across two genes of interest, *tufA* and *tufB*, in control and NMM-treated conditions. Each line is an insertion with the height reflecting the number of reads within a given insertion.

To better understand the effects of stabilized G4s in *E. coli*, Tn-seq was used to identify genes that alter cell growth in the presence of the G4 stabilizer, NMM. NMM is a porphyrin that can stack atop G4 structures, leading to stabilized G4s that can act as barriers to cellular processes such as DNA replication, transcription, or translation (Fig. 1A) [1, 26]. We predicted that strains with transposon insertions in genes that help withstand G4-stabilized conditions would be selected against when grown in the presence of NMM. In contrast, strains with insertions in genes that impair growth in G4-stabilized conditions would be positively selected (Fig. 1B). An initial screen using a transposon insertion library made in *E. coli* MG1655 (∼200 000 individual strains) revealed that insertions in *tolC*, which encodes a component of two major efflux systems [53], sensitized cells to NMM (Supplementary Fig. S1). These data suggested that TolC efflux can export NMM from the *E. coli* cytoplasm, which agreed with previous findings that loss of *tolC* in *E. coli* causes cytoplasmic accumulation of porphyrins [54, 55].

To facilitate cytoplasmic NMM retention in cells, a Δ*tolC E. coli* strain was used in a second set of Tn-seq experiments. Δ*tolC E. coli* transposon insertion mutants (∼500 000 total) were generated and grown ± NMM to obtain ∼1.5 million colonies from each growth condition. The positions and abundance of transposon insertions within the populations were identified by sequencing. Normalized weighted reads ratios were determined for each gene based on transposon insertion tolerance in control and NMM-treated growth conditions and gene length [46]. Positive or negative log_10_(normalized weighted reads ratio) (log_10_(n.w.r.r.)) values corresponded to genes in which transposon insertion is selected against or for, respectively, in NMM-treated conditions compared to control growth conditions. Of the 4312 genes assessed, transposon insertions were mapped in 3900, with 2210 genes containing an average of five or more unique hits in the control library, indicating strong coverage of transposon insertions across the libraries.

Comparison of transposon insertion tolerance ± NMM revealed the impact of gene disruptions in each growth condition (Fig. 1C and Supplementary Fig. S2). Measured log_10_(n.w.r.r.) values ranged from 3.70 to −4.78, with 577 genes having values ≥ 1.5 or ≤ −1.5, reflecting a wide-ranging impact of NMM on transposon selection. To systematically identify pathways that are most impacted by transposon insertions by stabilized G4s, gene ontology (GO) term analyses were carried out using genes with log_10_(n.w.r.r.) ≥ 1.5 or ≤ −1.5 (Supplementary Tables S1 & S2). The analysis indicated that disruptions in various pathways impact growth, positively or negatively, in the presence of NMM. Consistent with previous findings [26], *recA* and *rep*, which encode proteins involved in homologous recombination and accessory helicase activity, respectively, were conditionally important genes with log_10_(n.w.r.r.) values of 1.98 and 1.19. In contrast, insertions in genes with GO terms related to ribosome assembly or translation regulation were strongly enriched in cells grown in G4-stabilizing conditions, consistent with perturbation of translation aiding growth when G4s are stabilized. For example, GO term analysis revealed that of the 86 genes found to have ≤−1.5 log_10_(n.w.r.r.) values, terms related to translation, such as ribosome small subunit assembly and translation, were enriched 15.97-fold and 4.73-fold, respectively, with several other translation-related terms having fold-enrichment values within that range (Supplementary Table S2). Additionally, in the GO term analysis assessing genes with log_10_(n.w.r.r.) ≥1.5, cytoplasmic translation was under-represented, with <0.01-fold enrichment for genes annotated with this term.

Two genes from the screen, *tufA* and *tufB*, collectively contained ∼10% of the weighted reads in NMM growth conditions and had log_10_(n.w.r.r.) of −3.52 and −3.19, respectively (Fig. 1C and D), indicating a strong connection between their disruption and improved growth in the presence of the NMM (Supplementary Fig. S2). *tufA* and *tufB* insertion also aided growth in the pilot Tn-Seq screen with *tolC^+^* cells, with log_10_(n.w.r.r.) of −0.53 and −0.75, respectively (Supplementary Fig. S2). These striking results led to a deeper investigation into *tufA* and *tufB*, both of which encode the same protein (described further below).

**Figure 2.**
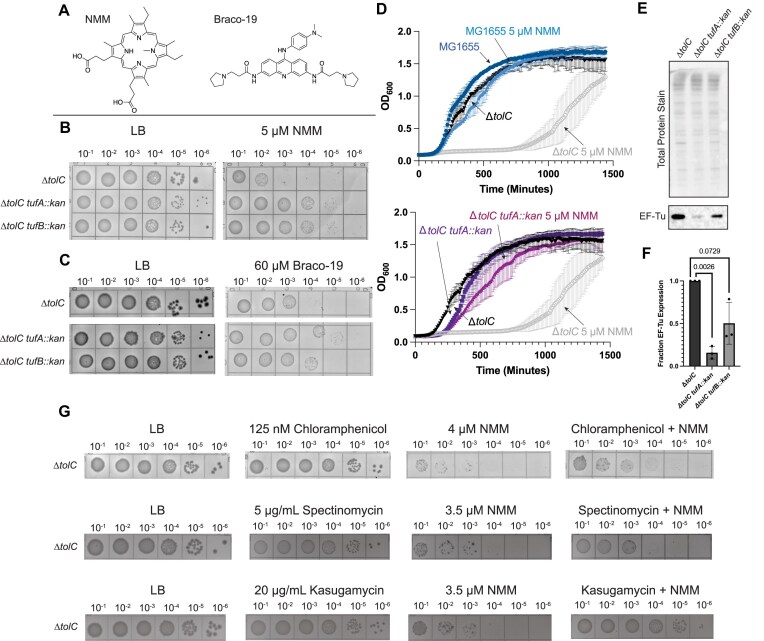
*tufA* and *tufB* deletions suppress growth defects in G4-stabilizing conditions. (**A**) Structures of NMM and Braco-19, two structurally distinct G4 stabilizers used in this study. (**B**) Spot plate experiments for Δ*tolC*, Δ*tolC tufA::kan*, and Δ*tolC tufB::kan* strains grown on Luria Broth (LB) or LB supplemented with 5 μM NMM. Each grid shows a 10-fold culture dilution, starting with cells plated at an OD_600 nm_ of 0.1. (**C**) Spot plate experiments as shown in (**B**) but grown in the presence and absence of 60 μM Braco-19. (**D**) Growth curves for MG1655, Δ*tolC*, and Δ*tolC tufA::kan*, grown in the presence and absence of 5 μM NMM. (**E**) Western blot and total protein stain of Δ*tolC*, Δ*tolC tufA::kan*, and Δ*tolC tufB::kan* blotting for EF-Tu. (**F**) Quantification of EF-Tu levels for *tufA* and *tufB* deletion compared to Δ*tolC* cells. (**G**) Top: Spot dilution plates plating MG1655 Δ*tolC* cells on LB-agar containing NMM, chloramphenicol, or both NMM and chloramphenicol. Middle: Spot dilution plates plating MG1655 Δ*tolC* cells on LB-agar containing NMM, spectinomycin, or both NMM and spectinomycin. Bottom: Spot dilution plates plating MG1655 Δ*tolC* cells on LB-agar containing NMM, kasugamycin, or both NMM and kasugamycin.

To validate the impact of *tufA* or *tufB* deletion in desensitizing *E. coli* to NMM, *tufA* or *tufB* deletion strains were generated in a Δ*tolC* background. As anticipated from the Tn-seq results, the plating efficiencies of the Δ*tolC tufA::kan* and Δ*tolC tufB::kan* strains were greater on NMM media than the Δ*tolC* parent strain, forming colonies at a 1000-fold more dilute culture than the Δ*tolC* control strain (Fig. 2A and B). To determine if this effect was due to the G4-stabilization properties of NMM, plating on a second medium containing a structurally distinct non-porphyrin G4 stabilizer, Braco-19, was measured. The Δ*tolC tufA::kan* and Δ*tolC tufB::kan* strains once again plated with higher efficiencies in the presence of Braco-19 than the Δ*tolC* control, albeit with lesser suppression than observed with NMM (Fig. 2A and C). Recent reports have demonstrated that NMM stabilizes RNA G4s to a greater extent than DNA G4s, whereas Braco-19 preferentially binds DNA over RNA G4s, which could be related to the modest difference in sensitivity to NMM and Braco-19 [28, 56]. Our analysis of hits from the Tn-seq data included *ypjD* (log_10_(n.w.r.r.) = −2.98), which encodes an uncharacterized membrane protein. Unlike observations with the Δ*tolC tufA::kan* and Δ*tolC tufB::kan* strains, Δ*tolC ypjD::kan* cells were resistant to NMM but remained sensitive to Braco-19 (Supplementary Fig. S2). Thus, the effects of *ypjD* mutation were specific to NMM, which may mean that the gene is involved in porphyrin import, export, or another aspect of porphyrin biology rather than activities with G4-stabilized nucleic acid structures, which is a shared property of NMM and Braco-19. This result highlights the strength of using structurally distinct G4 stabilizers to distinguish potential G4-specific effects (effects elicited by both) from compound-specific effects.

Growth in liquid media was also examined ± NMM for MG1655 (*tolC^+^*), Δ*tolC*, Δ*tolC tufA::kan*, and Δ*tolC tufB::kan* strains (Fig. 2D and Supplementary Fig. S2). Growth of the Δ*tolC* control strain was significantly delayed in the presence of NMM, and this delay increased with higher concentrations of NMM. Consistent with the plating results, the NMM-dependent lag phase was effectively eliminated by deleting either *tufA* or *tufB* (Fig. 2D). To determine if slow growth alone increased viability in the presence of NMM, the plating efficiencies of Δ*tolC*, Δ*tolC tufA::kan*, and Δ*tolC tufB::kan* strains were examined on M9 minimal medium ± NMM (Supplementary Fig. S2F). NMM retained a negative impact on growth for the Δ*tolC* cells, and increased plating efficiencies were observed for *tufA* and *tufB* deletion strains in the presence of NMM, indicating that slow growth alone does not explain increased viability in *tufA* or *tufB* deletion strains. Collectively, these data indicate that deletion of either *tufA or tufB* desensitized *E. coli* to the effects of G4 stabilizers.

### Modulating translation elongation and initiation attenuates the effect of G4 stabilizing compounds

*tufA* and *tufB* both encode for EF Tu (EF-Tu), an essential protein that escorts charged tRNAs to the ribosome during translation elongation [34, 57, 58]. There are ∼350 000 copies of EF-Tu in *E. coli*, making it the most abundant protein in the cell [34, 58]. EF-Tu-mediated delivery of aminoacylated tRNAs to the ribosome is thought to help govern the rate of translation elongation in *E. coli* [58]. Previous work has demonstrated that deletion of *tufA* leads to decreased cell growth (also observed in Fig. 2D) and protein synthesis rates [35, 36]. We hypothesized that deletion of *tufA* or *tufB* would lead to reduced levels of EF-Tu in cells, which could alter translation elongation and contribute to cellular resistance to G4 stabilization.

Quantitative western blot analysis of Δ*tolC*, Δ*tolC tufA::kan*, and Δ*tolC tufB::kan E. coli* strains demonstrated that deletion of either *tufA* or *tufB* led to decreases in relative EF-Tu levels, with the *tufA* deletion having the more significant impact (∼10-fold decrease in EF-Tu levels) (Fig. 2E and F). Thus, a reduction in EF-Tu levels is correlated with the ability of the cell to grow in G4-stabilizing conditions. However, direct evidence that altering translation elongation through *tufA* or *tufB* deletion desensitizes cells to the effects of G4 stabilizers was lacking.

To further test the idea that reduced translation rates allow *E. coli* to overcome G4 stabilizers, we next asked whether interfering with translation through a non-genetic intervention would improve cell growth in the presence of G4 stabilizers. This possibility was tested by plating cells on media containing NMM and sublethal doses of chloramphenicol or spectinomycin, which both interfere with bacterial translation elongation [59–61]. Adding either chloramphenicol or spectinomycin improved plating efficiency in the presence of NMM (Fig. 2G). Additionally, use of a translation initiation inhibitor, kasugamycin, strongly repressed the adverse effects of NMM (Fig. 2G) [62]. Thus, translation elongation or initiation inhibitors can relieve the adverse effects of NMM.

### CRISPRi screen reveals that impeding translation termination exacerbates the effects of G4 stabilization

Because Tn-seq relies on gene disruptions, the approach cannot identify roles for essential genes in G4 stabilizing conditions. We, therefore, used an IPTG-inducible CRISPRi method [39] to examine the effects of individually reducing the levels of 536 targeted genes (primarily essential genes) on cell growth ± NMM. The screen identified twelve genes that when targeted by CRISPRi machinery, sensitized cells to NMM, including genes involved in liposaccharide biosynthesis and transport (*lpxD, lpxB, lptE, lptC, lptA*), tRNA ligase activity (*leuS, aspS, valS*), protoporphyrinogen IX biosynthetic processes (*hemA*), cell division (*ftsZ*), and, notably, in translation elongation and termination (*fusA* and *prfA*) (Supplementary Fig. S3). We were motivated to investigate *fusA* and *prfA* as our Tn-seq screen had already shown that G4-stabilizers impact translation. EF G (EF-G, encoded by *fusA*) aids ribosome translocation and recycling and is involved in a ribosome rescue pathway [63, 64]. Release factor 1 (RF1, encoded by *prfA*) is involved in initiating translation termination at UAG and UAA stop codons [63].

**Figure 3.**
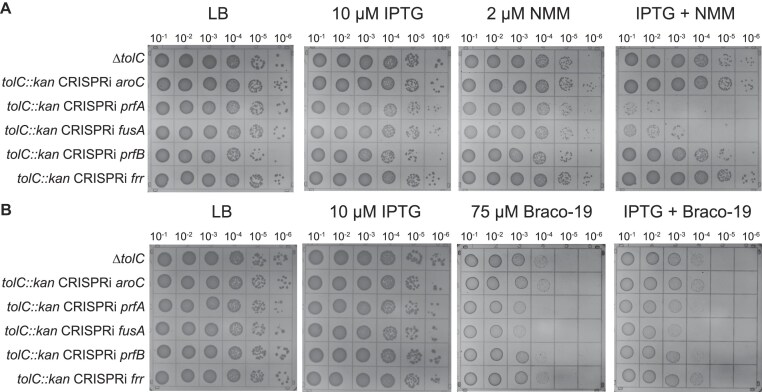
*prfA* and *fusA* knockdowns sensitize cells to G4 stabilizers. (**A**) LB-agar spot dilution plates in the presence of IPTG (induce CRISPRi machinery), NMM, or both to assess impacts of knockdown strains on growth in G4-stabilizing conditions (**B**) Same as (**A**) with Braco-19 as G4 stabilizing compound.

To characterize the effects of *prfA* and *fusA* knockdowns with G4 stabilizers, *tolC* deletions were generated in strains containing the IPTG-inducible CRISPRi machinery targeted to *prfA* and *fusA*. Control strains that lacked CRISPRi or in which CRISPRi targeted a control gene (*aroC*) that was not sensitized to NMM in the first screen were also tested. The strains were plated on media that included IPTG, NMM, or both IPTG and NMM to assess how knockdowns of RF1 and EF-G impacted *E. coli* growth (Fig. 3A). Both Δ*tolC* CRISPRi *prfA* and Δ*tolC* CRISPRi *fusA* strains grew poorly in the presence of IPTG and NMM compared to the Δ*tolC* and the Δ*tolC* CRISPRi *aroC* control strains (Fig. 3A). These findings were mirrored in a background with competent efflux pumps (*tolC^+^*) (Supplementary Fig. S4A).

To test whether the growth defects of the Δ*tolC prfA* or Δ*tolC fusA* knockdown strains were specific to NMM or more broadly observed with a structurally distinct G4 stabilizer, the strains were also grown in the presence of Braco-19 (Fig. 3B). These strains plated less efficiently than the control strains on Braco-19, although the addition of IPTG did not exacerbate the effects of Braco-19 on growth. This likely indicates that “leaky” expression of the CRISPRi machinery targeting *prfA* or *fusA* causes a mild fitness defect on Braco-19 alone. Nonetheless, the reduced plating efficiency of Δ*tolC* CRISPRi *prfA* and Δ*tolC* CRISPRi *fusA* strains indicate that the strains have a mild fitness defect in the presence of Braco-19 compared to the control strains, consistent with the defects being related to chemical stabilization of G4s.

Given the importance of *prfA* and *fusA* in suppressing the effects of G4 stabilizers, we explored the impact of two additional essential translation termination genes, *prfB* and *rrf*. Release factor 2 (RF2, encoded by *prfB*) initiates translation termination at UGA and UAA codons [63] and ribosome recycling factor (RRF, encoded by *rrf*) is involved in ribosome recycling in conjunction with EF-G [63]. Unlike *prfA* and *fusA*, CRISPRi-targeted knockdown of neither *prfB* nor *rrf* impacted *E. coli* cell plating efficiency on NMM or Braco-19 (Fig. 3). Thus, the effects observed with *prfA* and *fusA* were specific.

### Reduced expression of translation initiation factors rescues growth in G4-stabilizing conditions

Given that the antibiotic kasugamycin allows for enhanced cell viability in the presence of NMM (Fig. 2), we next tested if using the CRISPRi machinery targeted to translation initiation factors (*infA, infB*, and *infC*, encoding initiation factors (IF) 1, 2, and 3, respectively) had the same impact on growth as kasugamycin. Indeed, reduced expression of translation initiation factors led to better growth in the presence of NMM compared to control strains, with a more significant effect on growth with knockdowns of *infA* and *infB* than *infC* (Supplementary Fig. S4B). Thus, slowing translation initiation chemically or through genetic perturbations facilitates growth in the presence of NMM.

### Proteomic analysis reveals translation-related proteins are significantly altered in response to G4 stabilizers

The importance of translation factors in overcoming chemicals that stabilize G4s led to the question of how *E. coli* cells generally respond to such conditions. As a first step in addressing this question, a proteomic analysis was carried out to measure the effects of NMM on the levels of individual proteins in *E. coli*. Protein levels from early log-phase cultures of Δ*tolC* and Δ*tolC tufA::kan* strains grown ± NMM were measured to assess how NMM and reduced EF-Tu levels impacted expression. A total of 2509 proteins were detected in the dataset, approaching the limit of total proteins detected in previous *E. coli* proteomic studies [65, 66] (Supplementary Fig. S5). Additionally, the replicates correlated well as assessed by principal component analysis and Pearson Correlation coefficients (Supplementary Fig. S5).

**Figure 4.**
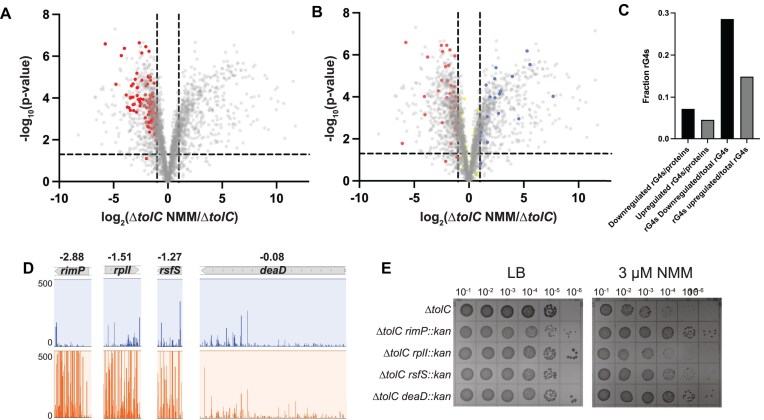
Proteomic profiles of Δ*tolC* in the presence and absence of G4 stabilizer. (**A** and **B**) Volcano plot of differences in individual protein levels between Δ*tolC* cells grown in the presence and absence of G4 stabilizer. Horizontal line indicates *P*-value of 0.05 and vertical lines indicate a 2-fold increase or decrease in protein levels. (**A**) Translation and ribosome biogenesis/assembly proteins that are downregulated in the presence of G4 stabilizer are depicted in red, opaque circles. (**B**) RNA G4s mapped onto the dataset, with red, opaque circles showing G4s in downregulated proteins, blue, opaque circles showing G4s in upregulated proteins, and yellow, opaque circles showing proteins that are not significantly changed in the proteomics experiment. (**C**) Quantification of the number of RNA G4s found in transcripts of proteins that are downregulated in the presence of NMM divided by total downregulated proteins and G4s found in proteins upregulated in the presence of NMM divided by total upregulated proteins. Additional quantification of the number of G4s found in the upregulated or downregulated proteomics category divided by the total number of G4s identified in *E. coli* (72). (**D**) Tn-seq results of four genes that were identified from proteomic analysis as proteins downregulated in the presence of NMM. Log_10_(ratio weighted reads) values are included above each transposon insertion profile. (**E**) Spot dilution plates of Δ*tolC* and Δ*tolC* strains harboring the gene deletions shown in (**D**) in the absence (left) or presence (right) of NMM.

GO term analyses were carried out for all proteins that were changed at least 2-fold in abundance when comparing two sample conditions (Supplementary Tables S3-S9). Comparing each dataset, the most significant overall difference was observed between the Δ*tolC* and NMM-treated Δ*tolC* cultures (Fig. 4 and Supplementary Fig. S6). Hundreds of protein levels were increased or decreased at least 2-fold in the presence of the G4-stabilizing compound. GO analyses carried out for proteins with statistically significant (as determined via q-value) ≥2-fold changes in the Δ*tolC* strain ± NMM revealed that many of the significantly impacted pathways were related to translation, ribosome assembly, and ribosome biogenesis (Fig. 4A, Supplementary Tables S3 & S4)). For example, ribosomal large subunit assembly and translation GO terms were 6.47 and 4.32-fold enriched, respectively, in proteins ≥ 2-fold less abundant in the Δ*tolC* NMM cultures compared to Δ*tolC* without NMM (Supplementary Table S4). This striking trend led us to investigate the overlap between GO clusters for genes that had increased levels of insertions under NMM-growth conditions in the Tn-seq experiment (GO terms identified in Supplementary Table S1) and those that are downregulated in the presence of NMM. This analysis revealed many overlapping GO terms, including several terms associated with translation, ribosome assembly, and protein–RNA complex assembly (Supplementary Table S5).

To better understand the impact of reductions in protein levels of translation factors, four non-essential translation-related proteins found to be downregulated in the presence of NMM were selected for further analysis: RimP, RplI, RsfS, and DeaD [67–70]. These proteins are either associated with ribosome maturation or bind directly to the ribosome. Each is significantly less abundant in Δ*tolC* cells grown in the presence of NMM than in control conditions. Genes for three of these proteins, *rimP*, *rplI*, and *rsfS*, tolerated higher levels of insertions in the NMM-treated conditions than in control growth conditions. In contrast, the level of transposon insertions in the fourth, *deaD*, was nearly the same in the two conditions (Fig. 4D). However, DeaD was one of the most downregulated proteins in the proteomic analysis, with a 54-fold decrease in expression in G4 stabilizing conditions. Δ*tolC deaD::kan*, Δ*tolC rimP::kan*, Δ*tolC rplI::kan*, and Δ*tolC rsfS::kan* strains were generated and grown ± NMM. Two strains (Δ*tolC deaD::kan* and Δ*tolC rimP::kan*) plated significantly more efficiently than the Δ*tolC* control strain in the presence of the G4 stabilizer NMM. In contrast, *tolC rsfS::kan* plated modestly more efficiently than the Δ*tolC* control (Fig. 4E). These findings further bolster the idea that altering translation processes aids growth in G4-stabilizing conditions.

Since *tufA::kan* suppresses the adverse effects of NMM, we predicted that the presence or absence of NMM would have little impact on gene expression within this strain. Indeed, comparing the Δ*tolC tufA::kan*± NMM revealed only one protein (Spy, a chaperone [71]) that was differentially detected via mass spectrometry in these samples. This aligns well with our initial finding that Δ*tolC tufA::kan* cells are not sensitized to G4-stabilizing conditions.

### Mapping RNA G4s to the Δ*tolC*± NMM proteomic dataset

One model that could explain why certain protein levels were reduced in the presence of NMM is that NMM-stabilized G4s within their transcripts impeded translation. Previously, 168 G4-forming sequences were found within the coding sequences for proteins in *E. coli* transcripts [72]. Mapping these elements onto the Δ*tolC*± NMM proteomic results revealed that 28.6% of the G4s were found in transcripts encoding proteins with reduced levels in the presence of NMM compared to 14.9% of G4s found in proteins with increased levels in G4-stabilizing conditions (Fig. 4). Thus, when G4s were present in transcripts of affected proteins, the protein levels were ∼2x more likely to be reduced than increased. There was no connection between the positions of RNA G4s in the open reading frame and the effects on protein abundance (Supplementary Fig. S6F). The remaining G4-containing element mapped to transcripts for proteins that were not found in the proteomic dataset or for which significant changes were not detected.

### Transcripts of several translation-related genes are less abundant in NMM-treated conditions

**Figure 5.**
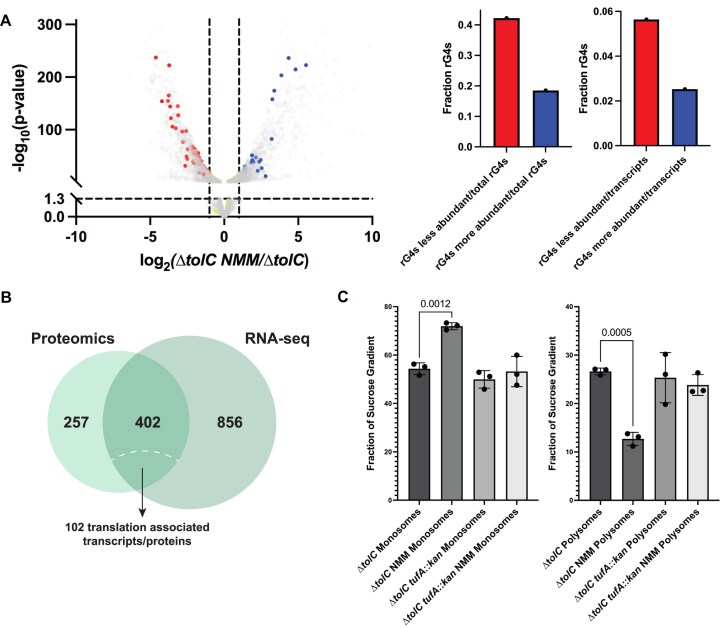
RNA-seq and sucrose gradient analysis of ribosomes reveal changes in translation in G4-stabilizing conditions. (**A**) RNA G4 forming sequences mapped onto the transcriptomics volcano plot for Δ*tolC*± NMM, with red, opaque points indicating transcripts less abundant in the presence of NMM that contain RNA G4s and blue, opaque points indicate transcripts that are more abundant in the presence of NMM and contain RNA G4s. Yellow points indicate transcripts not significantly altered by the presence of NMM and contain RNA G4s. Right: quantification of RNA G4s that were contained in more or less abundant transcripts shown as a fraction of total RNA G4s (left) and as a fraction of total upregulated or downregulated transcripts, respectively (right). (**B**) Overlap of proteins and transcripts identified as less abundant in the presence of NMM. (**C**) Quantification of the fraction of the AUC for monosomes and polysomes for Δ*tolC* and Δ*tolC tufA::kan*± NMM cultures.

After determining proteome changes in each growth condition, we next sought to identify if changes in protein abundance were due to differences in transcript abundance. RNA-seq was used to measure transcript levels differences in Δ*tolC* and Δ*tolC tufA::kan* cells grown ± NMM. RNA from cells grown to mid-log phase was sequenced using an Illumina platform targeting 20 million paired-end reads. Each sample had similar normalized gene expression levels via DeSeq2 analysis (Supplementary Fig. S7) [51]. Changes in transcript levels were considered biologically and statistically significant, with a ≥2-fold change in transcript detection and a statistically significant adjusted *P*-value of ≤ 0.05. Hundreds of significant transcript level differences were observed between cultures of (*1*) Δ*tolC* cells ± NMM, (*2*) Δ*tolC* cells + NMM and Δ*tolC tufA::kan* cells + NMM, and (*3*) Δ*tolC* and Δ*tolC tufA::kan* cells (Figs 5, Supplementary Fig. S7C, and Supplementary Tables S10-S15). As observed in the proteomic analysis, far fewer transcriptome changes were observed between the Δ*tolC tufA::kan*± NMM samples, with only one transcript (*mqo*) being less abundantly detected in Δ*tolC tufA::kan*+ NMM. While several transcripts were more abundant in Δ*tolC tufA::kan*+ NMM than cells grown without NMM, many of these transcripts mapped to genes associated with porphyrin biology (Supplementary Table S16), likely an off-target effect of NMM.

For Δ*tolC* cells ± NMM, many of the transcripts that were less abundant in the presence of NMM encode proteins involved in translation (Supplementary Table S11). Comparing the Δ*tolC* NMM transcriptomic and proteomic results, 402 genes had decreased transcript and protein levels in Δ*tolC* + NMM compared to Δ*tolC* (Fig. 5B). Among these, over 100 are broadly involved in translation, indicating that at both the transcript and protein levels, gene expression is altered in response to NMM.

We next assessed whether RNA G-quadruplex forming sequences correlated to Δ*tolC*± NMM transcripts level differences. Seventy-one RNA G4s were present in transcripts with reduced levels in the + NMM condition, 31 RNA G4s were found in transcripts with increased levels in the + NMM condition, and 66 were found in transcripts that were not significantly changed (Fig. 5A). Interestingly, this is different from what was observed for the proteomic dataset, where most of the RNA G4s mapped to proteins that were not significantly changed in the Δ*tolC*± NMM dataset comparison (Fig. 4). NMM may stabilize RNA G4s in these transcripts, leading to either degradation of the transcript or decreased translation of the implicated gene. However, decreases in transcript levels do not necessarily lead to a rapid decrease in the associated protein levels, denoting a more nuanced relationship between transcript and protein levels that could stem from cellular protein stability and bacterial mRNA instability.

### Global ribosome occupancy is altered in the presence of NMM

Finally, we examined whether ribosome occupancy on transcripts was altered in the presence and absence of NMM. Sucrose gradients were used to to analyze ribosome occupancy from Δ*tolC* cells ± NMM and Δ*tolC tufA::kan*± NMM. The monosome fraction in Δ*tolC* NMM cells was significantly increased compared to all other samples (Fig. 5C and Supplementary Fig. S8). This was matched with a subsequent decrease in the polysome fraction (Fig. 5C). In contrast, small and large subunit fractions were within experimental error for all conditions tested (Supplementary Fig. S8). NMM-treated samples from *tufA::kan* cultures matched those from untreated controls, indicating that a reduction of EF-Tu levels attenuates the difference in monosome/polysome ratio. The ratio change suggests that translation is significantly altered by stabilized G4s, which aligns with the proteomic and transcriptomic data.

In the Tn-seq dataset, genes encoding several ribosome rescue proteins (*arfA, arfB, hflX*, and *smpB*, [63, 64]) were identified as modestly important in G4-stabilizing conditions (Supplementary Fig. S9). We, therefore, sought to test if deletion of these genes exacerbated the effects of G4-stabilization, as ribosome rescue could be important for ribosomes stalled at RNA G4s. However, deletions of these genes or *smrB*, a recently identified gene important in ribosome collision prevention [73], did not produce cells with altered NMM sensitivity (Supplementary Fig. S9), indicating that their impact on G4 resistance is less pronounced than other translation factors.

## Discussion

RNA and DNA G4s form cellular regulatory elements in all domains of life. As G4 stabilization and *in vivo* detection tools have advanced, many studies have focused on the effects of G4s in eukaryotic systems. However, the roles of G4s in bacteria have received less attention [1]. To better understand the impact of G4 structures on bacteria, we have used chemical-genetic approaches to probe how disruption of genes in *E. coli* affects growth under conditions where transient G4 structures are chemically stabilized. Our work broadly showed that for *E. coli* grown in the presence of the G4-stabilizing compounds, disruptions of translation initiation, translation elongation, and ribosome assembly improved growth, whereas disrupting translation termination impaired growth. Transposon insertions in *tufA* of *tufB* genes, both encoding EF-Tu, were strongly selected for in G4-stabilizing conditions, whereas CRISPRi-mediated suppression of RF1 or EF-G sensitized cells to the same conditions. Analysis of protein and transcript levels revealed that downregulation of translation factors occurs in G4-stabilizing conditions at both transcript and protein levels. Chemical perturbation of translation elongation and initiation improved cell growth in the presence of the G4 stabilizer NMM. The results collectively suggest that by altering translation initiation or elongation, *E. coli* can combat the adverse effects of stabilized G4s and that disrupting translation termination sensitizes *E. coli* to G4-stabilizing agents. Because NMM stabilizes RNA G4s to a greater extent than DNA G4s [28, 56], these results point to RNA G4s as a potential regulatory feature in bacteria and a target for disrupting bacterial growth and fitness.

The discovery that altering the levels of multiple translation proteins in *E. coli* affects sensitivity to G4-stabilizing compounds suggests that RNA G4 inhibition of protein synthesis underlies impaired growth in the presence of the G4 stabilizers. *tufA* or *tufB* deletion reduces EF-Tu levels in *E. coli*, which is correlated with enhanced cell growth in the presence of either of two structurally distinct G4 stabilizers. EF-Tu brings aminoacylated tRNAs to the ribosome during translation elongation, a function proposed to be the rate-limiting step of translation and modulate cell growth rates [34–36, 58]. In contrast, CRISPRi suppression of RF1 or EF-G levels led to decreased viability in the presence of NMM and, to a lesser extent, Braco-19. RF1 initiates translation termination at stop codons UAG and UAA and is implicated in ribosome rescue [63]. Interestingly, RF2, which recognizes stop codons UGA and UAA and is implicated in a ribosome rescue pathway, was not conditionally important in G4-stabilizing conditions [63, 64]. The knockdown sensitivity of RF1 and not RF2 could be due to residue substitution in RF2 (A246T) present in *E. coli* K-12 strains that causes reduced recognition of the UAA stop codon by RF2, making RF1 the major release factor to recognize UAA [74, 75]. This decrease in translation termination efficiency by RF2 could be linked to the conditional importance of RF1 in G4-stabilizing conditions.

EF-G is involved in various functions, including ribosome recycling following translation termination, ribosome translocation during translation and recycling in ribosome rescue [63]. The role of EF-G in translocation is likely not linked to overcoming stabilized G4s, as we observed that the addition of the translation elongation inhibitor, spectinomycin, led to increased growth in NMM conditions. Spectinomycin inhibits translation elongation by interfering with ribosome translocation [61]. Instead, the role of EF-G in ribosome recycling or a ribosome rescue pathway is likely more important for overcoming stabilized G4s.

Analysis of NMM-induced changes at the protein and transcript level revealed significant effects in which ribosome assembly, biogenesis, and translation are downregulated (Figs 4 and 5). These pathways included genes selected for transposon insertion under NMM growth conditions. Moreover, treatment with NMM perturbed the polysome/monosome ratio in Δ*tolC* cells. These results reinforce the notion that an overall reduction in translation improves growth in G4-stabilizing conditions.

Although the results presented here connect G4 stabilization and translation, perturbations to several ribosome rescue factors did not impact *E. coli* viability in G4-stabilizing conditions (Supplementary Fig. S9). This could be due to redundancy across ribosome rescue pathways in *E. coli*, as there are three known ribosome rescue pathways that could clear stalled ribosomes [63]. It is also possible that RNA G4 structures themselves block efficient ribosome rescue for monosomes. Similar effects have been seen for ribosomes stalling at strong hairpin structures that may make ribosome A-sites less accessible for regulatory or rescue proteins [76]. It is possible that stabilized RNA G4s could similarly impede ribosome rescue factor access to ribosome A sites, preventing rescue of ribosomes from sites of RNA G4s and inducing the increase in the fraction of monosomes observed with NMM (Fig. 5). Such a model could help to explain how decreasing EF-Tu levels is beneficial for the cell, as lowering the pool of available EF-Tu would enhance ribosome A site accessibility for release/rescue.

RNA G4s in *E. coli* are all two-tetrad structures, which are less stable than three-tetrad RNA G4s examined in eukaryotes [1, 72]. *E. coli* may be sensitized to RNA G4 stabilization because they lack proteins that can efficiently unwind RNA G4s. Previous work found that inserting three-tetrad RNA G4s into *E. coli* was detrimental for growth [77]. *E. coli* may have selected against highly stable RNA G4s because of an inability to unwind RNA G4s generally or perhaps because RNA helicases in *E. coli* are unable to resolve three-tetrad RNA G4s. Nonetheless, two-tetrad G4s may play regulatory roles in *E. coli* without chemical stabilizers either when they transiently fold or when stabilized by G4-binding proteins.

**Figure 6.**
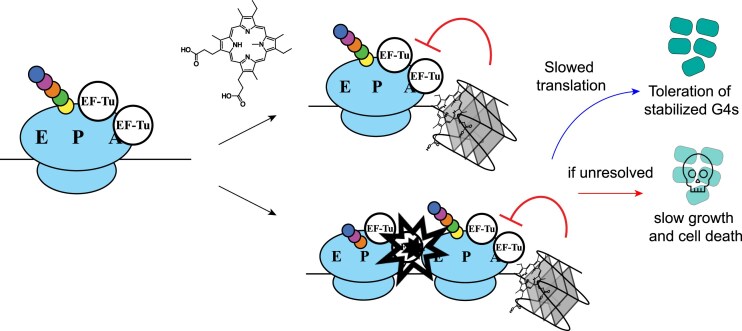
Model for RNA G4 disruption of growth in *E. coli*. NMM stabilization of RNA G4s could lead to either stalling upstream of the RNA G4 or lead to potential ribosome collisions upstream of the RNA G4. This is lethal or leads to impaired cell growth if unresolved, but slowed translation can increase tolerance for stabilized RNA G4s.

Models explaining how G4-stabilizing conditions could impact the growth of *E. coli* emerge from this study (Fig. 6). G4-stabilizing compounds are detrimental to growth in *E. coli*, and we reason that this is likely because they stabilize RNA G4s. While *E. coli* have helicases that can resolve DNA G4s, it appears less equipped to deal with stabilized RNA G4s. We have shown that slowing translation initiation or elongation or disrupting ribosome assembly leads to better outcomes for growth in NMM conditions. This indicates that it is beneficial for *E. coli* to lower the pool of ribosomes that will encounter RNA G4s, thus reducing possible collisions. Slower moving ribosomes or a lower pool of ribosomes could relieve the burden on ribosome rescue pathways that may inefficiently clear ribosomes stalled at RNA G4s. Specific translation termination and ribosome recycling factors (EF-G and RF1) become increasingly important in G4-stabilizing conditions, indicating their importance in clearing ribosomes at these sites of stalling. It is important to recognize that NMM or Braco-19 could also have off-target effects with stabilization of DNA G4 or with other cellular systems, which could impact *E. coli* growth. We have sought to limit such complications by using structurally-distinct G4 stabilizers in our study. Nonetheless, we note that transcription and translation are kinetically coupled in bacteria [78], which could result in a scenario where stabilized DNA G4s interfere with transcription, and subsequently, translation. However, the findings from our data point to translation as the major pathway impacted by G4 stabilization, which would be surprising if DNA G4s were the only target of NMM and Braco-19.

The results of this study provide new insights into the diverse roles of G4s in bacteria. Future chemical-genetic experiments could employ a range of G4-stabilizing compounds to identify common and unique pathways affected by each stabilizer. Related studies in other bacterial species and eukaryotes will also be important for determining whether translation suppression can be used to overcome stabilized G4 structures beyond *E. coli*. If such mechanisms are unique to bacteria, chemically induced G4-linked growth defects could form a novel basis for developing antibacterial agents. Further, future studies could investigate if altering translation similarly impacts how cells overcome other nucleic acid structures, such as hairpins and i-motifs.

## Supplementary Material

gkaf264_Supplemental_File

## Data Availability

Our RNA-seq is publicly available on GEO at https://www.ncbi.nlm.nih.gov/geo/query/acc.cgi?acc=GSE271718. Our Mass Spec data have been deposited to MassIVE (https://massive.ucsd.edu/ProteoSAFe/static/massive.jsp) under accession number MSV000095714. Our Tn-seq data are available through FigShare at: https://figshare.com/account/home#/projects/231767 and deposited to the Sequence Read Archive under project number PRJNA1115475. All additional raw data (including spot plate images and western blots) are available on Dryad (doi:10.5061/dryad.zcrjdfnn9).
